# Address of the Retiring President, Dr A. A. Blont

**Published:** 1865

**Authors:** 


### ADDRESS OF THE RETIRING PRESIDENT, DR. A. A. BLONT.

Read before the Mad River Valley Dental Association, April 27th, 1865.

We have come together once more, after a lapse of four years, which have been characterized by bloodshed and strife, and our once prosperous and happy country is now suffering the consequences of political and civil discord; and many a happy home, which once united in praise and thanksgiving around the family altar, is now desolated and constrained to mourn the loss of beloved ones who have offered up their lives as a sacrifice on the altar of their country's liberty.

While many of our profession have been engaged in the arduous duties of their calling, and laboring to advance the interests of our science, others again have devoted their time and energies to the service of their country. We have abundant cause to be thankful to an allwise Providence for continued health and prosperity, and for the rapid development of of that knowledge which is so essential to the usefulness of our profession, and for the prospect of a speedy termination of this civil war, and the overthrow of the enemies of our beloved country. Already, the "Rebellion is tottering to its fall," and but a little while the joyous tidings of peace will gladden every loyal heart.

But amidst all this terrible strife, our profession has not stood still, but steadily advanced from one improvement to another; and while such men as Taft, Atkinson, Watt and a host of others remain among us, we can never retrograde. One little instrument which has but recently been brought to the notice of our profession, is destined to revolutionize operative dentistry; and many teeth which heretofore were considered beyond the dentist's skill, will now be adorned with perfect fillings. To Dr. Wm. H. Atkinson belongs the honor of first introducing it to the notice of the profession, and a suitable testimonial should be awarded him for his valuable invention, and for his earnest and unceasing efforts to advance the interests of our science.

That instrument is the "Mallet," or "Common Gavel," which "Masonic tradition informs us is an instrument made use of by operative masons to break off the rough corners of stones, the better to fit them for the builder's use." But we, as operative dentists, are taught to make use of it for the more noble and glorious purpose of perfecting our fillings. For let me assure you, that the perfection which we all desire to attain can never be reached without the use of the mallet.

There are those among us who condemn its use, and call it a "merciless tool in the hands of adventurers;" but to such let me say, they but stand in their own light; for the time will soon come when they will be compelled to adopt its use, or be classed among those drones in all professions denominated "old fogys." Let me here say, that rather than relinquish the use of the mallet, I would abandon the profession; and I feel warranted in asserting that I but speak the sentiments of all who use it.

The Mad River Valley Dental Association, on the breaking out of the rebellion, was a prosperous society: consisting of some twenty members, who had banded together for the purpose of mutual instruction during the period of its existence, their labors were marked with unmistakable evidence of of success. Let what few of us who have come together to-day make an earnest effort to reorganize and sustain this society. There is no science within our knowledge which has made such rapid advancement as that of dentistry, and it can be traced almost solely to the influence of dental association. Let us, therefore, labor unceasingly to sustain the Mad River Valley Dental Association, and we will, in time, reap the reward of our own labors.

It is a common practice, upon the organization of dental societies, to adopt a "Fee Bill," which experience has taught us is a bad one; it has a tendency to keep out many who would willingly unite with some dental association, but who are too timid to take a bold stand in regard to their prices. It would be far better to let each one charge according as he values the services rendered. The better way to elevate the scale of dental fees is to educate the dentist; and as he increases in knowledge and skill, his charges will increase correspondingly. There is a class of dentists in our midst who gain notoriety and practice by advertisements of "cheap dentistry." Such men can not be prevailed upon to enter a society where they must subscribe to a fee bill; and another class still, who perambulate the country, from house to house, with their "kit" upon their back, and gain a miserable existence by flagrant untruth and sometimes downright dishonesty. Is it not better to bring such men within the influence of dental association, and, if possible, bring their minds to a proper appreciation of the calling which they had adopted: they bear the name of dentists, and what disgrace they bring upon themselves must reflect upon the profession.

Let us, by individual exertion, make the meetings of this society so interesting and instructive, that each member will feel that he has been benefited thereby; and let it be the constant aim of the association to disseminate light and knowledge, and to elevate the standard of dental science.

Since writing the above, the sad news of the death, by assassination, of our beloved President, Abraham Lincoln, has filled the land with deep, heartfelt grief, such as a nation seldom experiences.

But to the will of a just and merciful God, let us all, from the highest to the lowest in the land, with reverence bow.

